# TFEB, a potential therapeutic target for osteoarthritis via autophagy regulation

**DOI:** 10.1038/s41419-018-0909-y

**Published:** 2018-08-28

**Authors:** Gang Zheng, Yu Zhan, Xiaobin Li, Zongyou Pan, Fanghong Zheng, Zengjie Zhang, Yifei Zhou, Yaosen Wu, Xiangyang Wang, Weiyang Gao, Huazi Xu, Naifeng Tian, Xiaolei Zhang

**Affiliations:** 1 Department of Orthopaedics, The Second Affiliated Hospital and Yuying Children’s Hospital of Wenzhou Medical University, Wenzhou, 325000 Zhejiang Province China; 2 Zhejiang Provincial Key Laboratory of Orthpaedics, Wenzhou, 325000 Zhejiang Province China; 3 Department of Chemoradiation Oncology, The First Affiliated Hospital of Wenzhou Medical University, Wenzhou, 325000 Zhejiang Province China; 4 Department of Orthopaedics, The First Affiliated Hospital of Zhejiang University School of Medicine, Hangzhou, 310003 Zhejiang Province China; 5 The Second School of Medicine, Wenzhou Medical University, Wenzhou, 325000 Zhejiang Province China; 6 Chinese Orthopaedic Regenerative Medicine Society, Hong Kong, China

## Abstract

The blockage of autophagic flux in chondrocytes has been considered as a major reason for the excessive cellular apoptosis and senescence in osteoarthritis (OA) development; however, the molecular mechanism and therapeutic strategy for interrupted autophagic flux is still not clear. Most recently, the transcription factor EB (TFEB) is identified as a master regulator for autophagic flux via initiating the expression of multiple autophagy-related genes and lysosomal biogenesis. This research was performed to confirm whether TFEB expression and activity are impacted in OA development and to confirm the effect of genetic up-regulation of TFEB on autophagic flux and cellular protection in the in vitro and in vivo models of OA. We demonstrated that the expression and nuclear localization of TFEB is decreased in human and mouse OA cartilage as well as in tert-Butyl hydroperoxide (TBHP)-treated chondrocytes. Applying lentivirus to transfect chondrocytes, we found that TFEB overexpression rescues the TBHP-induced the autophagic flux damage, lysosome dysfunction and protects chondrocyte against TBHP induced apoptosis and senescence; these protections of TFEB are diminished by chloroquine-medicated autophagy inhibition. Our destabilized medial meniscus (DMM) mouse OA model shows that TFEB overexpression ameliorates the surgery-induced cartilage degradation, restrains the apoptosis and senescence of chondrocyte, and enhances the autophagic flux. In summary, our study indicates that the activity of TFEB in chondrocyte is involved in OA development, also TFEB overexpression may be a promising strategy for OA treatment.

## Introduction

Osteoarthritis (OA) is a common degenerative disease and is the fourth major cause of disability, but without any effective therapies^1^. Various factors such as age, adiposis, genetics, sexuality are considered to induce and aggravate OA development^2^. Despite multifold pathological changes associated with OA, the central hallmark of OA is still the structural destruction and dysfunction of articular cartilage^3^. As the only cell type in articular cartilage, chondrocyte could produce extracellular matrix molecules (ECM) including collagen and proteoglycans, the essential components for maintaining the function and structure of cartilage^4,5^. With the degradation of articular cartilage, the increasing inflammatory cytokines and oxidant stress induced the ROS production^6,7^; subsequently, ROS accumulation leads to the excessive apoptosis and senescence of chondrocytes^8,9^. Therefore, investigation of excrescent apoptosis and senescence of chondrocyte will contribute to a better understanding of cartilage degradation and new strategies for OA therapy.

It is widely accepted that autophagy is a protective mechanism through degrading the misfolded proteins and damaged organelles to maintain intracellular homeostasis and improve cellular survival and function^10^. Actually, autophagy is a dynamic process, in which subcellular membranes undergo structural changes, encapsulate cytoplasmic components, form autophagosomes and then fuse with lysosomes to form autolysosomes for degrading the contents^11^. This process is termed autophagic flux, which is also considered as a reliable indicator of autophagic activity. Numerous evidences showed autophagy plays a crucial role in OA development^12,13^, activating autophagy by local intra-articular injection of rapamycin alleviates cartilage degeneration in DMM mouse model^14^, demonstrating the protective role of autophagy in OA.

It has been reported that multiple process of autophagy is impaired in OA. The expression of Atg5 and LC3-II, the autophagosome membrane proteins, is reduced in human OA cartilage and also in surgery-induced OA dogs (model) and mice (DMM, destabilized medial meniscus)^15,16^, indicating autophagosome initiation process is impaired. In a more recent study, our group found that high accumulation of p62 (a protein representing the degradation level of autophagic vacuoles) appears in human OA cartilage and DMM mouse cartilage, suggesting that autophagic degradation is blocked in OA chondrocytes^17^. Moreover, Kim et al. observed accumulation of lysosomes and decline of lysosomal activity in human OA chondrocytes relative to normal chondrocytes, also they showed that destroying lysosome results in enhanced chondrocyte apoptosis and inhibition of autophagy^18^. Together, these studies suggest that targeting autophagosome initiation and lysosomal fitness might restore autophagy flux, which will in turn protects chondrocyte against cellular damage.

Transcription factor EB (TFEB) is a member of microphthalmia-associated transcription factor (MITF)/transcriptional factor E (TFE) family. Most recently, TFEB is identified as a master regulator of the autophagic flux via inducing lysosome biogenesis and promoting the autophagosome formation and fusion with lysosome^19–22^. And there is growing evidence that TFEB plays a crucial role in neurodegenerative diseases such as Alzheimer’s, Parkinson’s and Kennedy’s by accelerating the clearance of the accumulated proteins^23–26^. However, the effect of TFEB in OA is still no clear. In this article, we reported that TFEB activity is declined in OA, while TFEB overexpression could suppress the excessive apoptosis and senescence of chondrocyte in vivo and in vitro through the regulation of autophagy lysosome pathway (ALP).

## Results

### The expression and activation of TFEB is declined in human and mouse knee articular cartilage

**Fig. 1:**
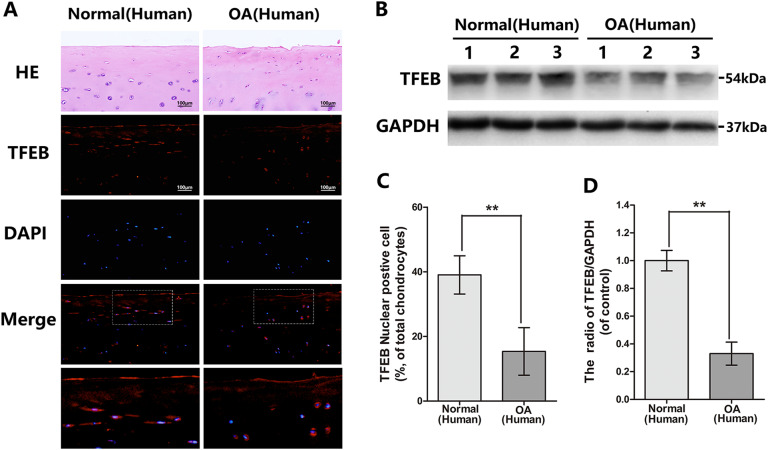
The TFEB level in normal and OA human knee articular cartilage. **a** Representative H&E staining and immunofluorescence staining of TFEB in human knee articular cartilage from normal and osteoarthritis patients (bar: 100 μm). **b**, **d** The protein expression of TFEB in human chondrocytes derived from normal and osteoarthritis patients. **c** Quantitation of immunofluorescence staining of TFEB in human knee articular cartilage from normal and osteoarthritis patients. All data represent mean ± S.D. (*n* = 5). **P < 0.01

**Fig. 2:**
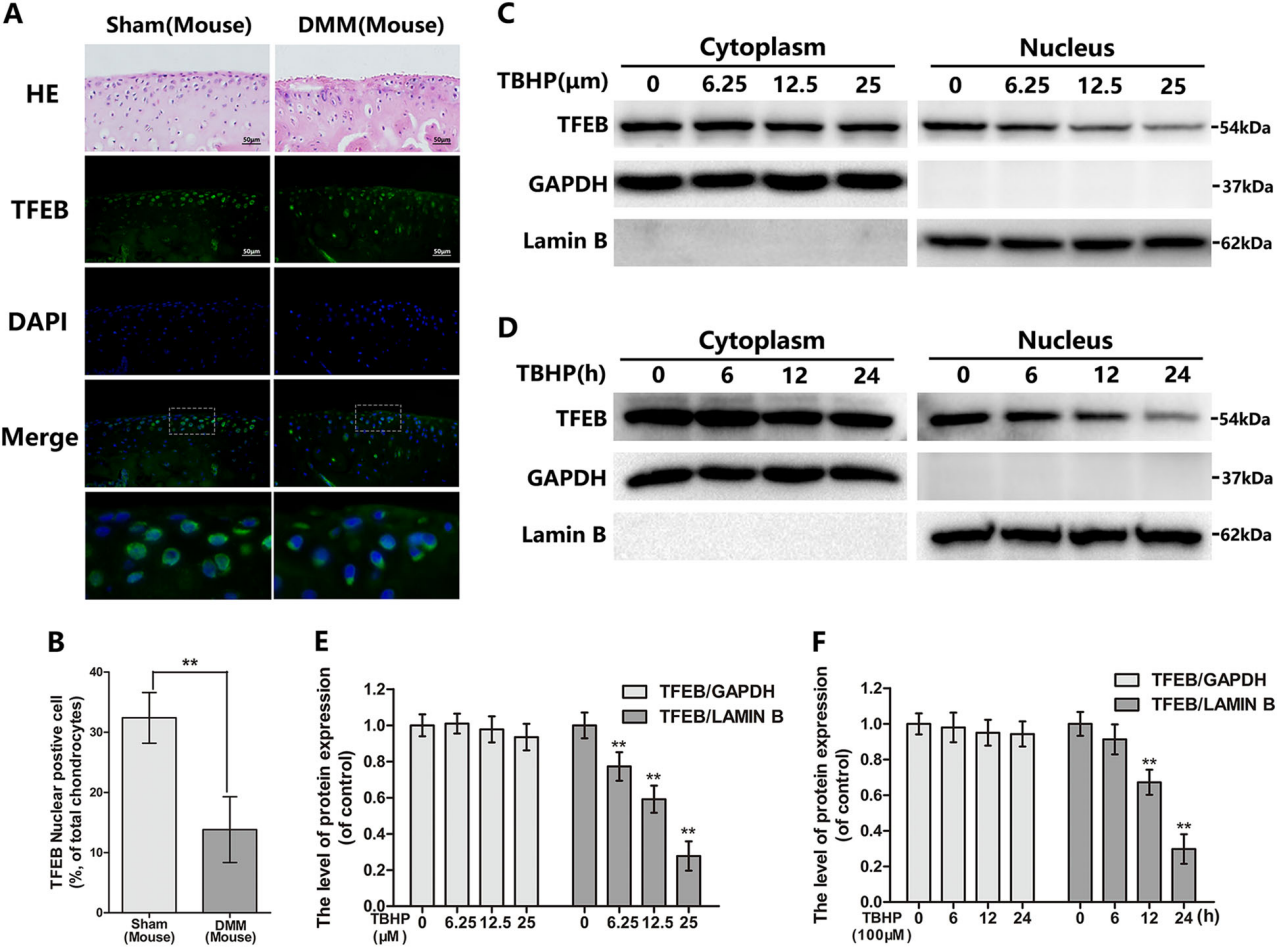
The nuclear expression of TFEB in mouse chondrocytes is decreased in OA model in vivo and in vitro. **a** Representative H&E staining and immunofluorescence staining of TFEB in mouse knee articular cartilage from sham and DMM model. (bar: 50 μm) **b** Quantitation of immunofluorescence staining of TFEB. **c**–**f** The protein expression of TFEB in cytoplasm and nuclear in mouse chondrocytes treated as above. All data represent mean ± S.D. (*n* = 5). **P < 0.01

To ascertain the change of TFEB level in OA development, immunofluorescence was applied to compare the TFEB nuclear translocation status in chondrocytes in normal and OA human articular cartilage. We found the percentage of TFEB nuclear positive chondrocytes reduced in the articular cartilage from OA patients (Fig. 1a, c). The same results were observed in sham and DMM mouse cartilage (Fig. 2a, b). In addition, we isolated primary human knee articular chondrocytes from normal and OA patient and researched by western blot. As shown in Fig. 1b, d, the TFEB expression declined in the OA derived chondrocytes relative to normal. Thus, we concluded OA leads to the down-regulation and inactivation of TFEB in chondrocytes.

### TBHP inhibits TFEB nuclear expression with a both time and concentration-dependent manner in chondrocytes

Oxidative stress participates in OA development. To explore molecular mechanism of TFEB, we exposed chondrocytes at Tert-Butyl hydroperoxide (TBHP) to mimic OA model in vitro, which is an external stimulant of oxidative stress characterized by more stable and sustained-release performance than H_2_O_2_. And CCK8 assay showed the chondrocyte viability is decreased after TBHP administration (Figure S1A and B). Western blot analysis revealed that TBHP restrained the TFEB nuclear expression with a both time and dose-dependent in chondrocytes (Fig. 2c–f). Interestingly, TFEB cytoplasm expression decreased in TBHP-treated chondrocytes, but it was not statistically significant. These results are basically consistent with studies in vivo.

### TFEB overexpression suppresses the oxidative stress-mediated premature senescence and apoptosis in chondrocyte

**Fig. 3:**
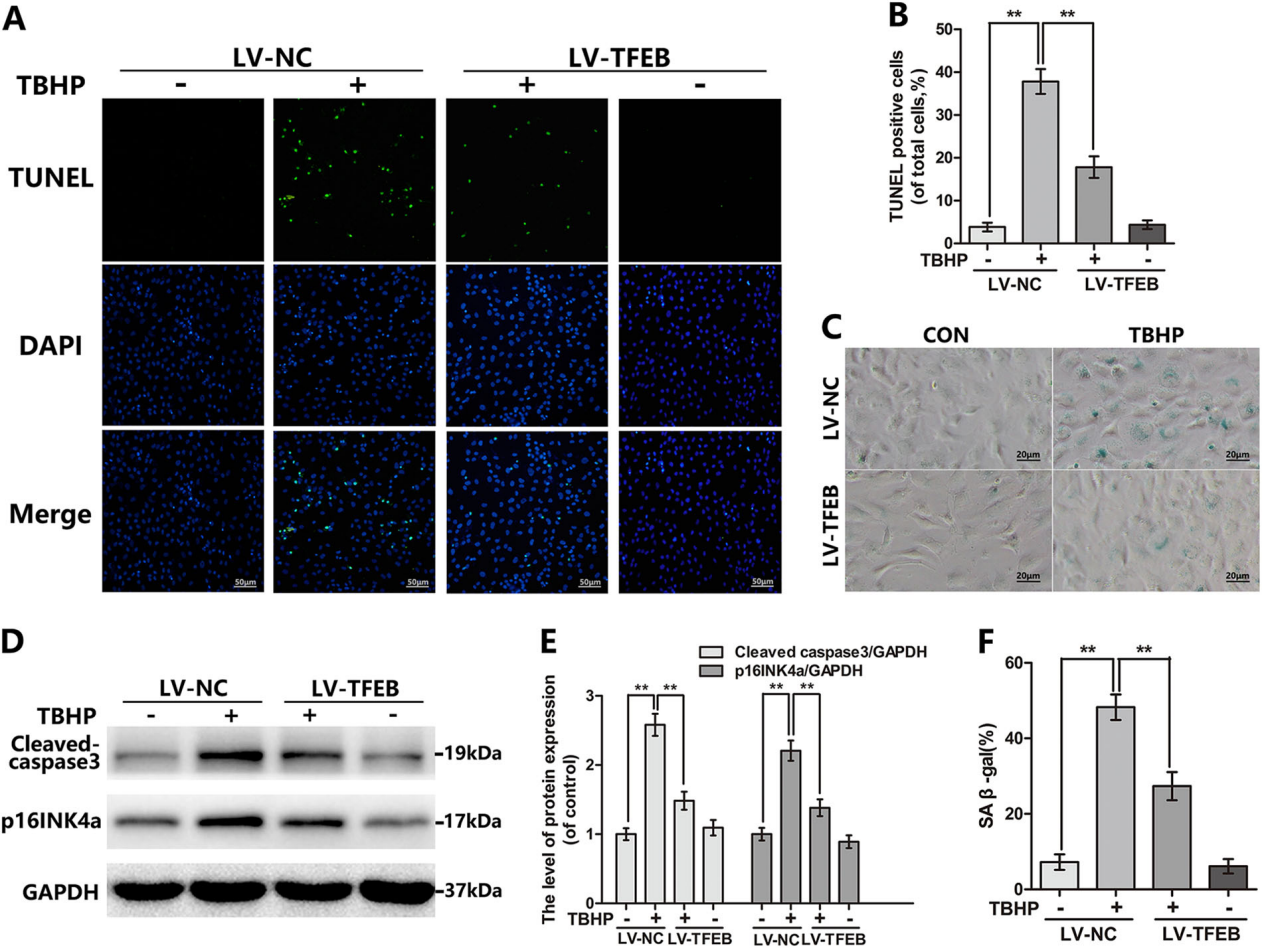
TFEB overexpression attenuates the TBHP-induced apoptosis and senescence in chondrocytes. **a**, **b** TUNEL staining assay was performed in chondrocytes as treated above (bar: 100 μm). **c**, **f** SA-β gal staining assay was performed in chondrocytes as treated above (bar: 20 μm). **d**, **e** The protein expression of Cleaved-caspase3 and p16INK4a in mouse chondrocytes as treated above. All data represent mean ± S.D. (*n* = 5). **P < 0.01

OA is an aging-related degenerative disease with chondrocyte senescence and apoptosis, which are potentially related to oxidative stress. To confirm the TFEB protection of chondrocyte under oxidative stress, chondrocytes were transfected with lentivirus-TFEB (LV-TFEB) to overexpress TFEB protein. The transfection efficiency was measured by western blot (Figure S2). The cellular senescence level is estimated by SA-β-gal staining and p16INK4a, a classical senescence marker protein. The TBHP-treated chondrocytes exerted higher SA-β-gal activity and p16INK4a protein level relative to the control group, whereas TFEB overexpression significantly prevents this increment (Fig. 3c–f). To make sure whether TBHP-induced apoptosis is alleviated by TFEB overexpression, the levels of the cleaved caspase 3 and DNA damage were detected by western blot and TUNEL staining respectively. We found that TFEB overexpression down-regulated the TBHP-induced increasing levels of the cleaved caspase 3 expression and TUNEL positive cells (Fig. 3a, b, d, e). However, silence TFEB in chondrocytes by siRNA enlarged the TBHP-simulated the upregulation of cleaved-caspase 3 and p16INK4a expression, suggesting the TFEB knockdown deteriorated the apoptosis and senescence of TBHP-exposed chondrocytes (Figure S3C and D). Better yet, the cell viability of chondrocytes with TFEB knockdown existed no obvious difference relative to the chondrocytes transfected by CON-siRNA. During the TBHP treatment, the TFEB silence in chondrocytes declined the cell viability compared to the control group (Figure S1C). These results indicate that the senescence and apoptosis caused by TBHP are attenuated following LV-TFEB transfection in chondrocyte.

### TFEB accommodates the autophagy lysosome pathway in chondrocytes

**Fig. 4:**
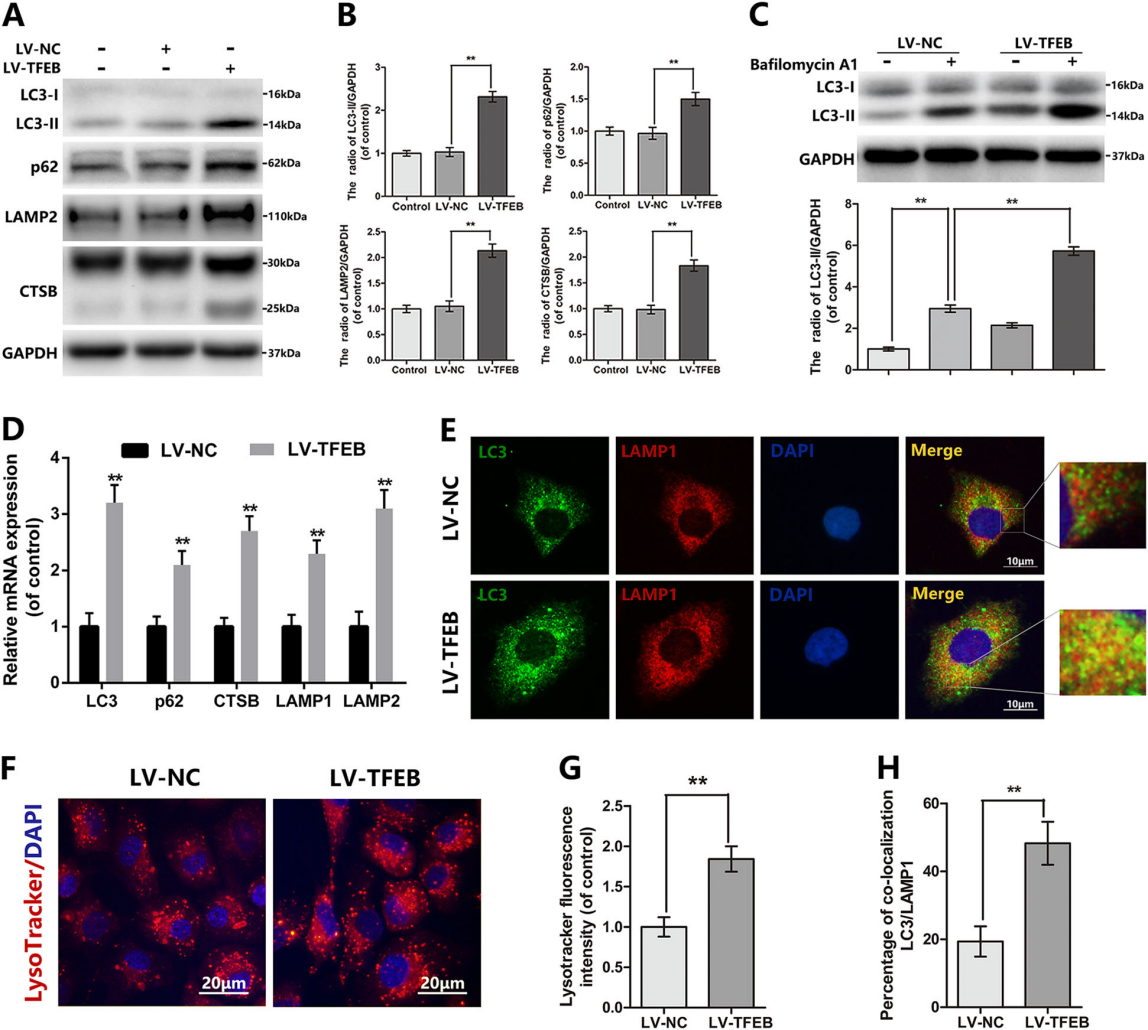
TFEB regulates autophagic flux in chondrocytes. **a**, **b** The protein expression of LC3, p62, LAMP2 and CTSB in mouse chondrocytes treated as above. **c** The protein expression of LC3 in mouse chondrocytes treated as above. **d** The mRNA expression of LC3, p62, CTSB, LAMP1, and LAMP2 in mouse chondrocytes treated as above. **e** Immunofluorescence double-labeled staining for co-localization of LC3 with LAMP1 in mouse chondrocytes treated as above (Green: LC3, red: LAMP1, bar: 10 μm). **f**, **g** The Lysotracker staining in mouse chondrocytes treated as above (bar: 20 μm). **h** The quantitation of the percentage of co-location of LC3/LAMP1 was detected by image J. All data represent mean ± S.D. (*n* = 5). **P < 0.01

Recent evidences in cell biology regard TFEB as a master controller of the ALP, but its function in chondrocyte has not been reported. First of all, overexpression of TFEB can increase the mRNA and protein levels of LC3, p62, CTSB, LAMP1, and LAMP2, which are both the TFEB target genes (Fig. 4a, b, d). The LC3-II level is a dynamic variation process in the autophagy, which is decreased with the degradation of lysosomes and increased autophagosome formation. And the difference of LC3-II expression level, between the chondrocytes are cultivated with bafilomycin A1 (a typical lysosomal inhibitor) and those without, is used to assess the activity of autophagic flux. Our results in Fig. 4c indicated the autophagic flux increased following TFEB overexpression. Conversely, the siRNA-induced TFEB down-regulation declines the level of the autophagic flux in chondrocyte (Figure S3B). Autolysosome generates from the fusion of autophagosome and lysosome, which is the final organelle to degrade internalized cargo. Therefore, the autophagosome-lysosome fusion and the lysosomal degradation function are the foundation and essential component element for the autophagic flux. By double immunofluorescence staining with LC3-II (a marker of autophagosome) and LAMP1 (a marker of lysosome), we observed that TFEB overexpression discernibly increased the autophagosome-lysosome fusion relative to the control group (Fig. 4e, h). LysoTracker Red (LTR) is a sensitive lysosomotropic pH probes, which appears red fluorescence in a pH-dependent manner. Compare to the LV-NC group, the cells transferred by LV-TFEB are accompanied by the increase in red fluorescence, indicating that TFEB overexpression promotes the lysosomal acidification in chondrocyte (Fig. 4f, g). In a word, all of the above results confirm that TFEB acts as a key role of ALP in chondrocyte.

### TBHP-induced autophagic flux blockage and lysosome dysfunction is alleviated by TFEB overexpression in chondrocytes

**Fig. 5:**
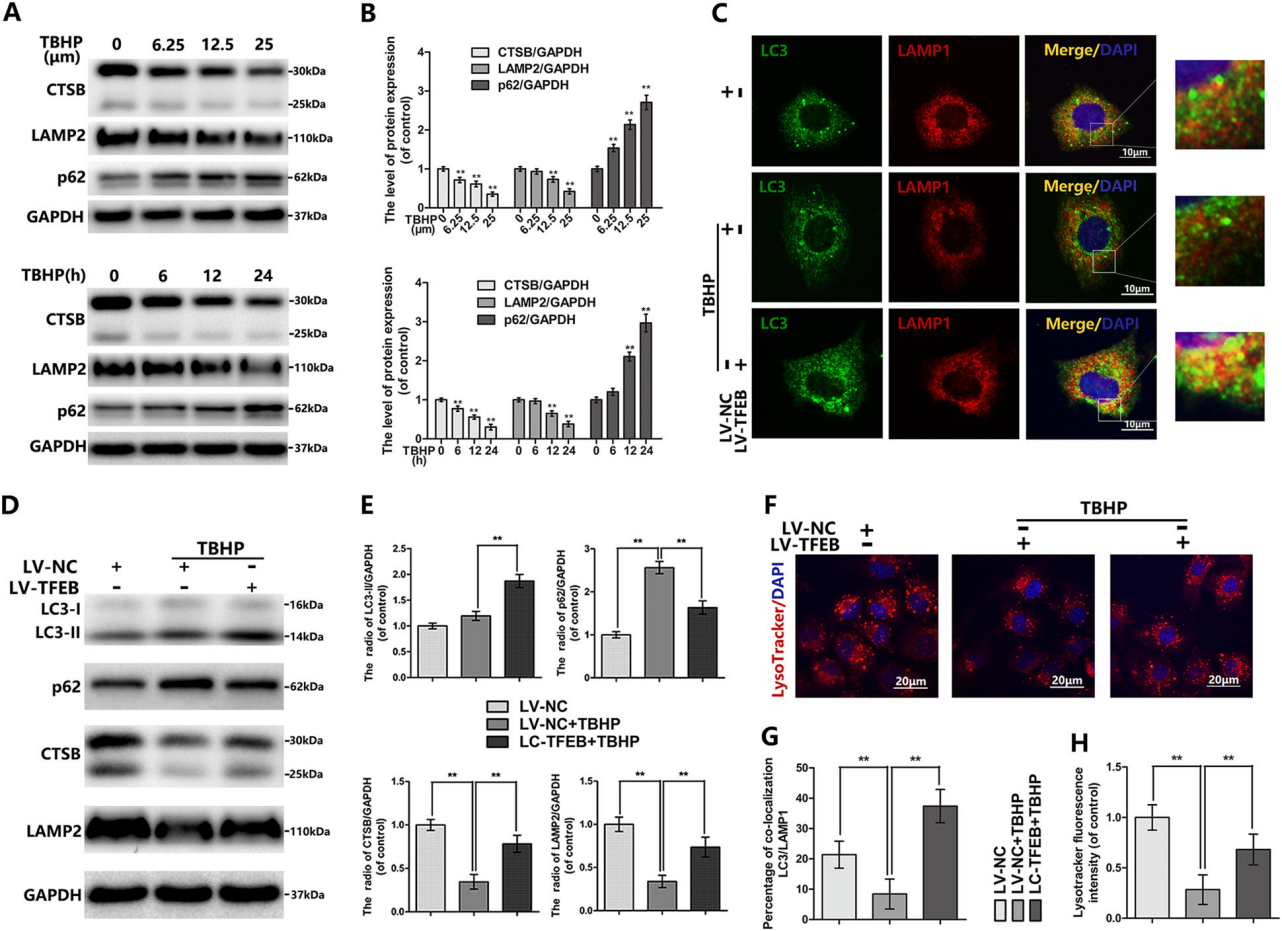
TFEB overexpression rescues the blockage of autophagic flux induced by TBHP in chondrocytes. **a**, **b** The protein expression of CTSB, LAMP2 and p62 in mouse chondrocytes treated as above. **c** The protein expression of LC3 in mouse chondrocytes treated as above. **d** Immunofluorescence double-labeled staining for co-localization of LC3 with LAMP1 in mouse chondrocytes treated as above (Green: LC3, red: LAMP1, bar: 10 μm). **d**, **e** The protein expression of LC3, p62, LAMP2 and CTSB in mouse chondrocytes treated as above. **f**, **h** The Lysotracker staining in mouse chondrocytes treated as above (bar: 20 μm). **g** The quantitation of the percentage of co-location of LC3/LAMP1 was detected by image J. All data represent mean ± S.D. (*n* = 5). **P < 0.01

Oxidative stress-induced autophagic changes is time and concentration-dependent. By western blot analysis we observed that TBHP reduced the expression of the CTSB, LAMP2 and increased accumulation of p62 in a dose and time-dependent manner (Fig. 5a, b). Next, we applied the following experiment to confirm whether TFEB overexpression can restore the ALP dysfunction in TBHP-exposed chondrocytes. Overexpression of TFEB reversed the TBHP-induced changes of the LC3-II, p62, CTSB and LAMP2 (Fig. 5d, e). Our double immunofluorescence staining demonstrated that the blockage of autophagy-lysosome fusion occurs in chondrocytes after TBHP treatment, whereas TFEB overexpression can dramatically relieve this performance (Fig. 5c, g). Simultaneously, overexpression of TFEB restored TBHP-inhibited lysosomal acidification confirmed by LTR staining (Fig. 5f, h).

### CQ reverses TFEB-induced protective effect in chondrocytes under the oxidative stress

**Fig. 6:**
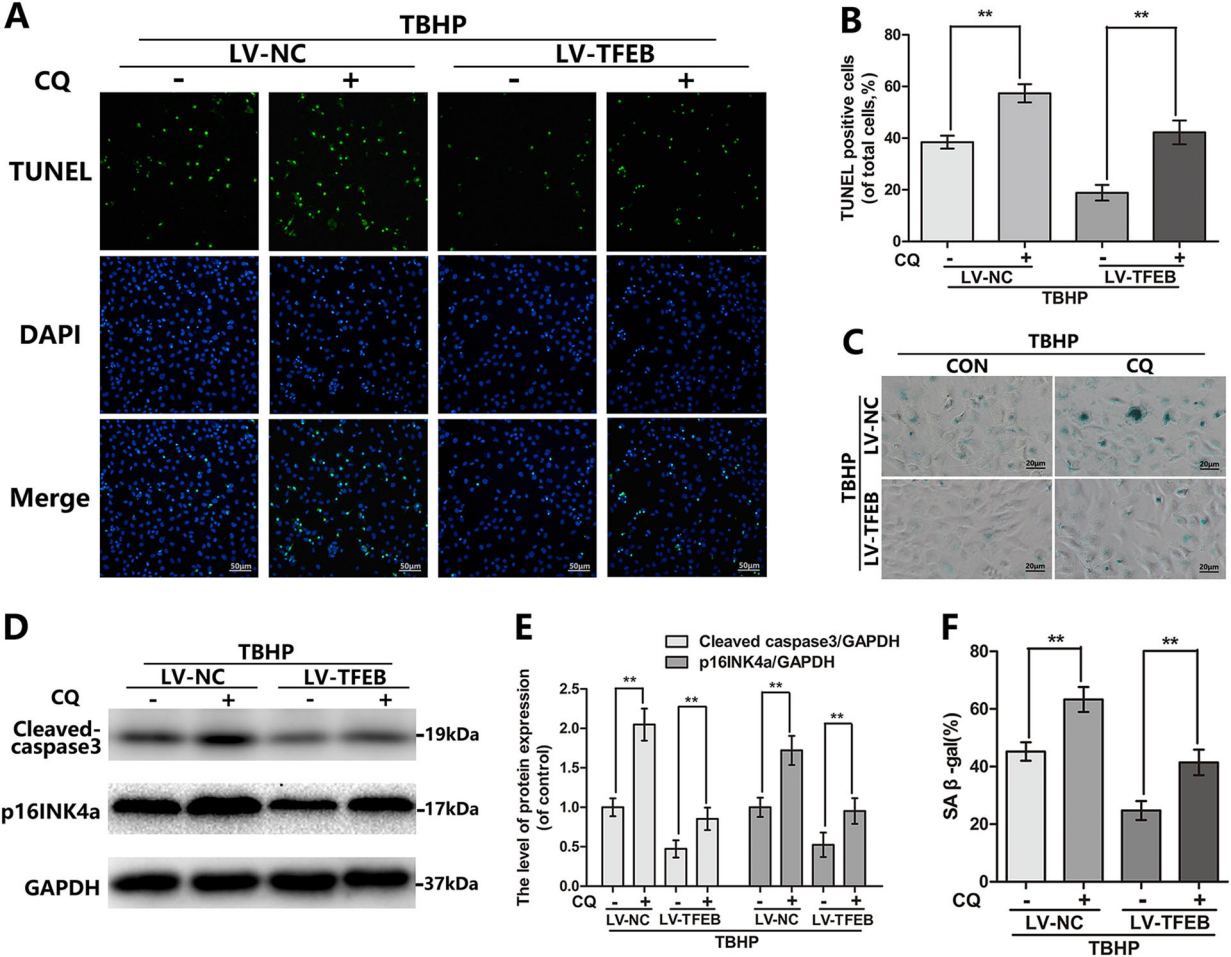
Chloroquine counteracts the protective effects of TFEB in TBHP-exposed chondrocytes. **a**, **b** TUNEL staining assay was performed in chondrocytes as treated above (bar: 100 μm). **c**, **f** SA-β gal staining assay was performed in chondrocytes as treated above (bar: 20 μm). **d**, **e** The protein expression of Cleaved-caspase3 and p16INK4a in mouse chondrocytes as treated above. All data represent mean ± S.D. (*n* = 5). **P < 0.01

Now it is generally accepted that autophagy is the positive character in cell under the stress. Interestingly, unlike the traditional autophagy agonists, TFEB overexpression not only mediates the formation of autophagosome but also up-regulates the biogenesis and function of lysosome to promote the degradation of autophagosome. Thus, we chose the chloroquine (CQ), a lysosomal cavity alkalizer, rather than the 3-Methyladenine (3-MA), to suppress the downstream of autophagic flux^11^. As shown in Fig. 6, the protection of TFEB overexpression against TBHP-based apoptosis and senescence including the reduction of the cleaved caspase 3 and p16INK4a protein levels, and the percentage of TUNEL positive cells and the SA-β-gal activity as performed by the SA-β-gal staining, are either markedly attenuated or completely abolished by co-treat the chondrocytes with CQ. These data confirmed that the protection of TFEB overexpression against apoptosis and senescence requires the unobstructed autophagic flux.

### TFEB regulated the expression of ECM-associated proteins via autophagy

To probe whether TFEB-controlled autophagy participates in the ECM metabolism in chondrocytes, we measured the collagen II and aggrecan by ELISA and MMP13 (the main matrix degrading enzyme) by immunofluorescence. As shown in figure S4, TFEB overexpression effectively suppressed the TBHP-induced increase of MMP13 and degeneration of collagen II and aggrecan, which is weakened or abolished after CQ treatment.

### TFEB overexpression ameliorates OA development in DMM mouse model

**Fig. 7:**
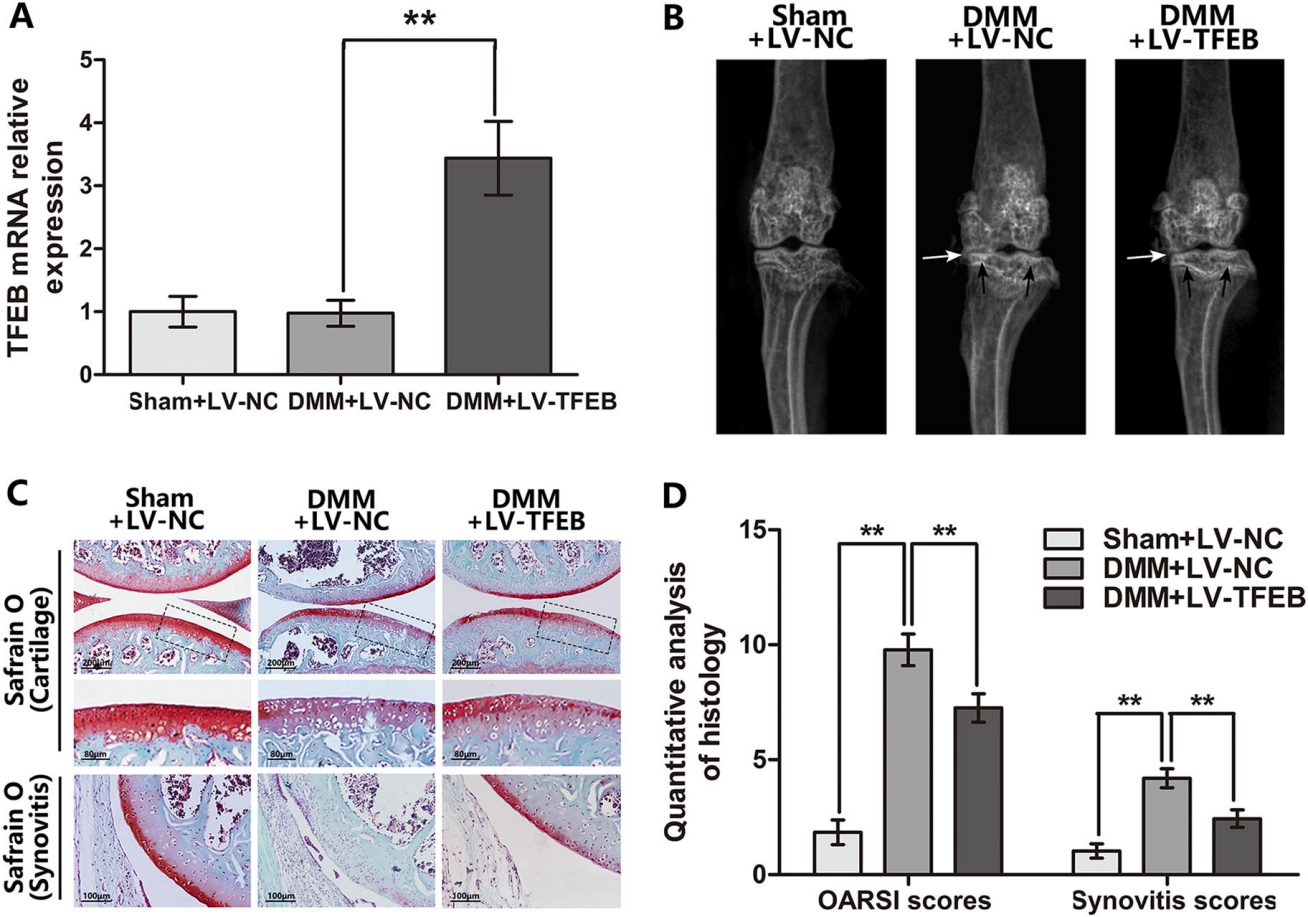
TFEB overexpression ameliorates OA development in mouse DMM model in vivo. **a** RT-PCR result showed lentivirus-mediated TFEB overexpression was successful at 2 weeks after lenti-virus injection (*n* = 5). **b** Digital X-ray image of mouse knee joints from different experimental groups. Narrowing of joint space was found in both OA and treatment group (white arrows), the calcification of cartilage surface was obviously shown in OA group (black arrows). **c** Representative S-O staining of cartilage and synovitis in three groups at 8 week post-surgery (bar: 200 or 100 μm). **d** OARIS scores of cartilage and the scores of synovitis in three groups. All data represent mean ± S.D. (*n* = 15). **P < 0.01

To investigate the potential therapeutic effects of TFEB overexpression in vivo, we injected the LV-TFEB into the knee joint of the mouse and performed X-ray and Safranin O staining to assess imageology and histomorphology differences of mouse knee joint. As shown in Fig. 7b, the joint space is aberrantly narrowed and cartilage surface density is increased after surgery. Nevertheless, milder calcification of cartilage surface and lower narrow of joint space was observed in DMM + LV-TFEB group. By Safranin O staining, we observed the erosion and hypocellularity of the superficial articular cartilage, and the loss of proteoglycan, and the thickening and hypercellularity of synovium in the DMM + LV-NC group at 2 months after surgery (Fig. 7c). In contrast, the DMM + LV-TFEB group shows the more complete cartilage surface, and the richer proteoglycan, and the thinner synovium relative to the DMM + LV-NC group. The results of the OARSI and synovitis scores are consisted with staining, forced expression of TFEB declines the OARSI and synovitis scores, which is risen in DMM + LV-NC group (Fig. 7d).

**Fig. 8:**
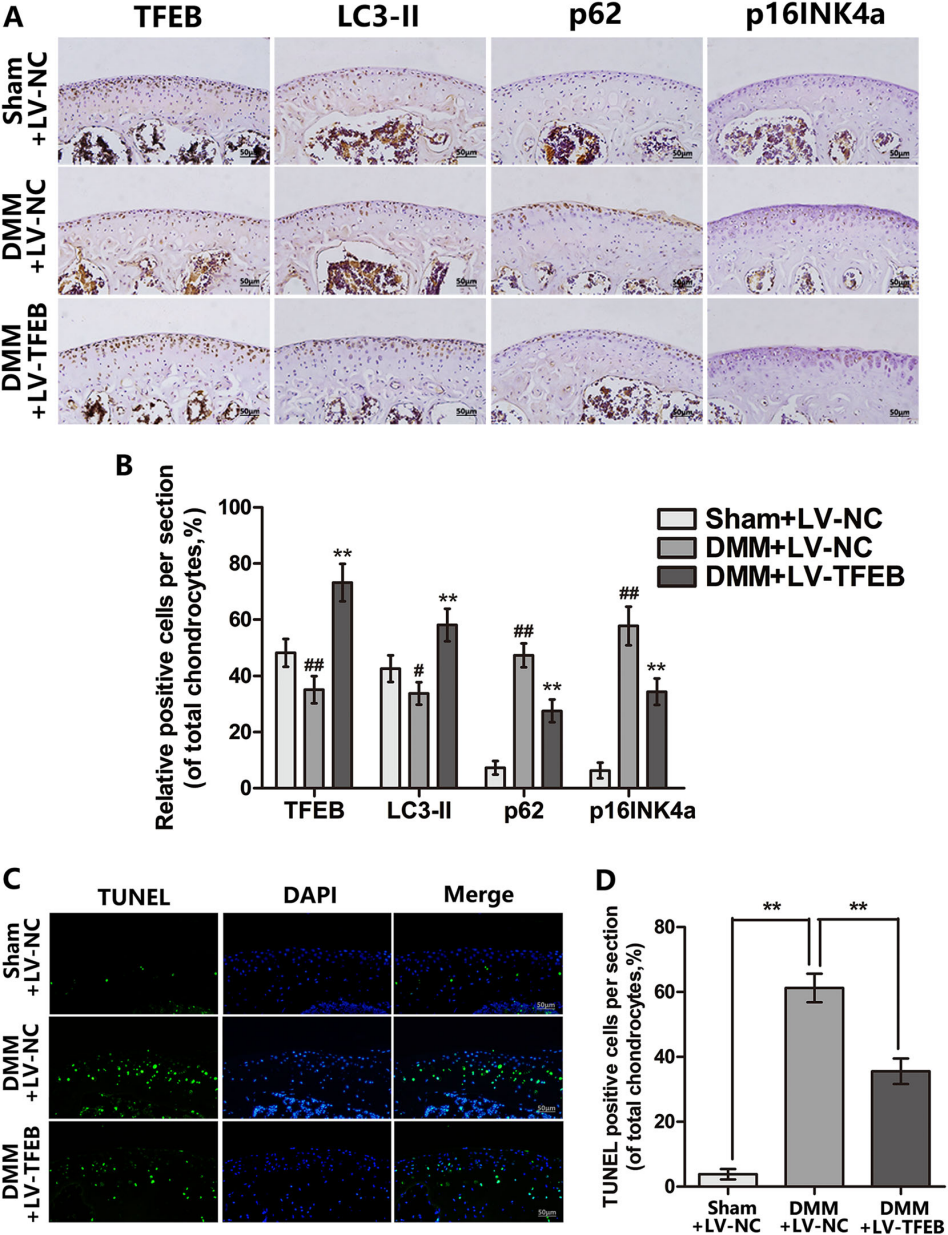
Protective effects of TFEB against apoptosis and senescence in chondrocyte is autophagy dependent. **a**, **b** Immunohistochemical staining assay of TFEB, LC3-II, p62 and p16INK4a in the mouse cartilage (bar: 50 μm). **c**, **d** TUNEL staining assay in the mouse cartilage. All data represent mean ± S.D. (*n* = 5). **P < 0.01, versus DMM + LV-NC, ^#^*P* < 0.05; ^##^*P* < 0.01, vs. Sham + LV-NC

In addition, the lentivirus transfection efficiency in mouse articular cartilage was confirmed by RT-PCR. The TFEB mRNA level in DMM + LV-TFEB group is increased in chondrocytes at 14 days after lentivirus injection (Fig. 7a). Meanwhile, compared to the DMM + LV-NC group, the more TFEB positive chondrocytes are presented at the mouse knee articular cartilage in the DMM + LV-TFEB group at 60 days after surgery by immunohistochemical staining (Fig. 8a).

### Overexpression TFEB improves autophagic flux and prevents apoptosis and senescence of chondrocytes in vivo

The apoptosis and senescence of chondrocytes were estimated by TUNEL staining and p16INK4a immunohistochemical staining respectively. And the experiment results showed that the higher proportion of cellular apoptosis and senescence in DMM + LV-NC group than the sham + LV-NC group, whereas the lentivirus-mediated TFEB overexpression reversed this pathological phenomenon (Fig. 8). In addition, we applied immunohistochemical staining of LC3-II and p62 to detect the TFEB-induced autophagy in vivo. As shown in Figs. 8a, b, the lentivirus-mediated TFEB overexpression could increase the LC3-II expression and decrease the DMM-induced p62 accumulation in mouse articular cartilage. These data implied promoting autophagic flux is a potential mechanism underlying the anti-apoptotic and senescent effect of TFEB in vivo.

## Discussion

Recently studies reported TFEB activity or expression is impaired in the process of several neurodegenerative disorders including Alzheimer’s, Parkinson’s^24,26–28^. And genetic overexpression or pharmacological activation of TFEB shows beneficial effects against these diseases development in the cellular and animal models^23–25,27,29^. However, it is still unknown whether TFEB involves in OA development. This study demonstrated the TFEB activity and expression are declined in human and mouse degenerative cartilage. To further elaborate whether TFEB plays a protective role in OA, we transfected chondrocytes with lentivirus to overexpress TFEB in vivo and in vitro. As a result, overexpression of TFEB obviously suppressed the apoptosis and senescence of chondrocyte and alleviated DMM-induced cartilage degeneration via rescuing the deficient ALP.

As a basic helix-loop-helix leucine-zipper (bHLH-Zip) transcriptional factors, TFEB activity and subcellular localization are dependent on the TFEB phosphorylation level^19,30^. In normal physiological conditions, TFEB is mainly phosphorylated by mechanistic target of rapamycin complex 1 (mTORC1) at multiple critical serine residues and bund with chaperone 14-3-3 and thereby sequestered at the cytoplasm^31–33^. Once mTORC1 activity is suppressed, the phosphatase calcineurin induces the TFEB dephosphorylation^34^. Then dephosphorylated TFEB transfers to the nucleus and directly binds to the promoter regions of various autophagy genes and also the palindromic E box (i.e., CACGTG), which named the coordinated lysosomal expression and regulation network, to regulate autophagy and lysosome biogenesis^19,22^. It is reported that mTORC1 is aberrantly activated in human OA cartilage and DMM-induced mouse OA model, which might explain our results about the decreased TFEB activity in OA^14,16^. In addition, either pharmacologically or genetically inhibition of mTORC1 activity attenuated OA pathology in animal models^14,16^. Meanwhile, cartilage-specific tuberous sclerosis complex 1 (Tsc1, mTORC1 upstream inhibitor) knockout (TSC1CKO) mice exhibited spontaneous OA with aberrant chondrocyte proliferation and hypertrophic differentiation^35^. These researches prompt that the declined activity of TFEB might be a consequence of aberrant mTORC1 activity in OA, which of course requires further experimental validation.

As the aging-associated degeneration diseases, the pathophysiology in OA development is characterized by multiple factors involvement, including inflammatory, mechanical and metabolic stressors, which all results in the increased level of reactive oxygen species (ROS)^36^. With excessive ROS production, the damaged DNA and outer membrane-destroyed mitochondria gradually cumulates in the nucleus and cytoplasm, respectively^37–39^. And the later leads to mitochondrial apoptotic proteins releasing such as cytochrome c (Cyt C), which triggers the caspases activation^40,41^. The disturbance between the ROS overproduction and antioxidant deficit, defined as oxidative stress, causes cellular apoptosis and accelerates senescence thought destroying telomerase stability^7,8,42^. In the present study, we used TBHP as ROS donor to stimulate the apoptosis and senescence of chondrocyte in in vitro study. And we found that TBHP-induced DNA damage, cleaved caspase3 and p16INK4a increasing, and SA-β-gal activation are decreased by TFEB overexpression, suggesting that TFEB overexpression suppresses the apoptosis and senescence of chondrocyte in conditions of oxidative stress.

Our study found that TFEB nuclear expression is reduced when chondrocytes are treated with TBHP; while some researchers reported that ROS could induce the TFEB nuclear translocation in other cell models, which seems to be contrary to our results^43–45^. These contradictory results may be caused by the variation in degree of ROS. The studies which showed ROS may induce TFEB nuclear translocation apply a different way to induce ROS than we do. They use a much higher concentration of TBHP (100 μM) for a duration of 4 h in their in vitro studies^20,46,47^, and this kind of acute stimulation may cause a self-protective responding to transient oxidative stress, which may drive TFEB to translocate to the nucleus; while we use a relative mild concentration of TBHP (25 μM) for total 24 h duration, which might be more able to simulate the pathological conditions in vivo.

Autophagic flux has been widely demonstrated as a potential therapeutic target for OA, as it may help to combat apoptosis and senescence^48^. Studies showed that the autophagosome formation is reduced, also lysosome activity is destructive in the process of OA. We showed here that TFEB overexpression may not only promote autophagosome formation (as shown by increased LC3-II expression in Fig. 4a), but also restore lysosome function (as shown by up-regulated CTSB expression and lysosomal acidity in Fig. 4a, f), and the fusion of autophagosome and lysosome (as shown by promoted co-localization of LC3-II/LAMP1 in Fig. 4e), which ultimately restores the TBHP-induced disruption of autophagic flux. In addition, our in vitro siRNA-medicated TFEB knockdown experiments reveal that downregulating TFEB leads to declined autophagic flux in chondrocytes, suggesting TFEB may indeed regulate autophagy flux in chondrocytes.

Besides autophagic flux regulation, TFEB also participates in various biological pathways^46,49^. To further confirm whether autophagic flux plays an indispensable role in protective effects of TFEB against apoptosis and senescence in TBHP-treated chondrocytes, CQ, which is a lysosomal cavity alkalizer and commonly used as the classical inhibitor of the downstream in autophagic flux, was used in our study. The results of western bolt, TUNEL and SA-β-gal staining demonstrated that CQ significantly attenuates the protection of TFEB in the cultured chondrocytes under TBHP-induced oxidative stress, suggesting TFEB exerts its protective effects mainly through autophagy flux regulation.

In conclusion, we firstly demonstrated that the expression and activation of TFEB is decreased in human OA cartilage and DMM-induced mouse OA cartilage. And, TFEB overexpression fortifies the autophagic flux and lysosome function, which contribute to protect chondrocyte again apoptosis and senescence stimulated by TBHP. In addition, our in vivo experiment confirmed that TFEB overexpression suspend the DMM-induced cartilage degradation, which is associated with the reestablish the autophagic flux. These data indicate that TFEB can be considered as a potential target for the treatment of osteoarthritis.

## Materials and methods

### Ethics statement

Human articular cartilage tissue collection and the experiments involved in human cartilage tissue was approved by Ethical Committee of the Second Affiliated Hospital, Wenzhou Medical University and following the guidelines of the Declaration of Helsinki^50^. The letter of ethics approval was provided in supplementary file 1. The experimental procedures and the animal use and care protocols were according to the Guide for the Care and Use of Laboratory Animals of the National Institutes of Health and was approved by the Animal Care and Use Committee of Wenzhou Medical University (ethic code: wydw2014-0129).

### Reagents and antibodies

Tert-Butyl hydroperoxide solution (TBHP), bafilomycin A1 (Baf), CQ and type II collagenases were purchased from Sigma-Aldrich (St Louis, MO, USA). Antibodies against TFEB were purchased from Proteintech (Rosemont, IL, USA). Antibodies against cleaved caspase 3, LC3, and CTSB were purchased from Cell Signaling Technology (Beverly, MA, USA). Antibodies against GAPDH, LAMP2, p16INK4a, p62, MMP13 and Lamin B were the products of Abcam (Cambridge, MA, USA).

### Human cartilage and chondrocytes culture

The normal human articular cartilages from 5 donors with no significant clinical and imaging features of OA were obtained from femoral condyles and tibial plateaus at autopsy (45–74 years old; mean, 56.7 years; Kellgren-Lawrence grade,^51^ 0 or I; *n* = 5). The OA human articular cartilages were obtained form 8 patients (51–77 years old; mean, 58.9 years; Kellgren–Lawrence grade, III or IV; *n* = 8) undergoing total knee arthroplasty (representative X-ray image was presented in Figure S5). Cartilage tissues were cut into 5-μm sagittal sections and embedded in paraffin for histological analysis. To obtain primary human chondrocytes, hyaline cartilage was cut into pieces and incubated with 2 mg/ml of collagenase II in DMEM/F12 at 37 °C for 4 h. After washing by PBS and resuspension, chondrocytes were cultured in a six-well plate at a seeding density of 2 × 10^5^ cells per ml in DMEM/F12 supplemented with 10% FBS and 1% antibiotic in 5% CO2 at 37 °C. Chondrocytes no later than first passage were used for the experiments.

### Primary mice chondrocytes culture

Ten immature C57BL/6 mice (5 males and 5 females, 10 days) were euthanized with an overdose of sodium pentobarbital. The knee cartilages of mice were collected carefully under aseptic conditions by a dissecting microscope, and the tissues were treated with 2 mg/ml (0.1%) collagenase II for 4 h at 37 °C. Next, the digested cartilage tissues were suspended and seeded into tissue culture flasks. The chondrocytes grow in DMEM/F12 (Gibco, Invitrogen, Grand Island, NY) with 10% fetal bovine serum (FBS; Hyclone, Thermo Scientifc, Logan, UT, USA) and 1% penicillin/streptomycin antibiotics (Gibco, Invitrogen, Grand Island, NY) in the incubator maintained at 5% CO_2_ at 37 °C. The medium was changed firstly after 24 h incubation. When up to 80% to 90% confluence, the cells were harvested by using 0.25% Trypsin-EDTA (Gibco, Invitrogen, Grand Island, NY). Then, cells were replanted into 10 cm culture plates at the appropriate density. The second-passage chondrocytes were used for all of our experiment due to no significant changes was noticed during cells passaging from passage 0 to passage 2. The chondrocytes were cultured in the incubator maintained at 5% CO2 at 37 °C and the complete medium was changed every other day.

### Cell treatment

To investigate the expression of TFEB level in chondrocyte during oxidative stress, chondrocytes were treated with different concentrations of TBHP (0, 6.25, 12.5, 25 µM) for 24 h and with the same concentration of TBHP (25 µM) for different time (0, 6, 12, 24 h). During performing autophagic flux assay, chondrocytes were treated with 100 nM bafilomycin A1 for 1 h. In order to determine the effect of the autophagic flux in TFEB-induced protection, cells were pretreated with 50 μm CQ (an autophagy inhibitor) for 6 h before the addition of TBHP.

### Lentivirus transfection

TFEB was overexpressed via transfection of Lenti-TFEB (GeneChem, Shanghai, China). The cells were transfected with Lenti-TFEB or Lenti-NC at a confluence of 30–50%; >95% of the cells were viable 12 h later. The medium was then changed, the cells incubated for a further 3 days, and passaged for further experiments. Transfection efficacies were measured via western blotting.

### siRNA transfection

siRNA for mouse TFEB gene was obtained from Santa Cruz Biotechnology (Dallas, TX, USA). Chondrocytes were seeded in a six-well plate and cultured for 24 h to 60–70% confluency. The cells were transfected with 50 nM negative control or siRNA duplexes using Lipofectamine 2000 siRNA transfection reagent (Thermo Fisher, UT, USA). After the following further treatments, cells were harvested for western blot experiments.

### SA-β-gal staining

The level of senescence was measured by senescence associated β-galactosidase (SA-β-gal) staining kit (Beyotime, Shanghai, China) according to the instruction. Aging chondrocytes showing higher SA-β-gal activity were stained blue.

### TUNEL staining

The level of DNA damage was detected by TUNEL staining. Chondrocytes or cartilage sections were fixed and then stained with in situ cell death detection kit (Roche, Basel, Switzerland) according to the manufacturer’s instructions for 30 min at 37 °C and the nuclei was stained with DAPI. Twenty-five fields of each slide were randomly selected and captured under a fluorescence microscope (Olympus Inc., Tokyo, Japan) to count TUNEL positive cells.

### LTR staining

LTR staining were applied to assess the numbers and function of lysosomes. Chondrocytes were stained with 50 nM LysoTrackerRed (Invitrogen, Grand Island, NY) for 30 min at 37 °C, and nuclei were stained with Hoechst 33258 for 10 min. Red fluorescence images of at least 25 random microscopic fields were acquired per slide for microscopic observation with a fluorescence microscope (Olympus Inc., Tokyo, Japan), and fluorescence intensity was measured using Image J (Bethesda, MD, USA).

### Real-time PCR

The total RNA was extracted from cartilage by TRIzol reagent (Invitrogen, Grand Island, NY). One microgram of total RNA was used to synthesize cDNA (MBI Fermantas, Germany). For the quantitative realtime PCR (qPCR), a total 10 μl of reaction volume was used, including 5 μl of 2 × SYBR Master Mix, 0.25 μl of each primer and 4.5 μl of diluted cDNA. Parameters of RT-PCR were: 10 min 95 °C, followed by 40 cycles of 15 s 95 °C and 1 min 60 °C. The reaction was performed using CFX96Real-Time PCR System (BioRad Laboratories, California, USA). The cycle threshold (Ct) values were collected and normalized to the level of GAPDH. The level of relative mRNA of each target gene was calculated by using the 2-ΔΔCt method. The primer sequences were as follow: for TFEB, Forward 5′-AAGGTTCGGGAGTATCTGTCTG-3′, Reverse 5′-GGGTTGGAGCTGATATGTAGCA-3′, for LC3, Forward 5′- CGTCCTGGACAAGACCAAGT-3′, Reverse 5′-ATTGCTGTCCCGAATGTCTC-3′, for p62, Forward 5′-GCTGAAGGAAGCTGCCCTAT-3′, Reverse 5′-TTGGTCTGTAGGAGCCTGGT-3′, for CTSB, Forward 5′- TGTGGTGGTCCTTGATCCTT-3′, Reverse 5′-AATCTGTCCAATGGTCGGGC-3′, for LAMP1, Forward 5′-AGCATACCGGTGTGTCAGTG-3′, Reverse 5′-GTTGGGGAAGGTCCATCCTG-3′, for LAMP2, Forward 5′-TGCTTTCTGTGTCTAGAGCGT-3′, Reverse 5′-CCTGAAAGACCAGCACCAACT-3′.

### Enzyme-linked immunosorbent assay (ELISA)

The concentration of Collagen II and Aggrecan in cell culture supernatants was detected by using commercial ELISA kits (Abcam, Cambridge, USA) according to the manufacturer’s instructions. All assays were performed in five times.

### Western blot analysis

The total protein extracted from chondrocytes was isolated using RIPA lysis buffer with 1 mM PMSF (Phenylmethanesulfonyl fluoride) and on the ice for 10 min followed by 15 min centrifugation at 12000 rpm and 4 °C, and then protein concentration was measured using the BCA protein assay kit (Beyotime, Shanghai, China). 40 ng of protein was separated by sodium dodecylsulfate-polyacrylamide gel electrophoresis and transferred to a polyvinylidene difluoride membrane (Bio-Rad, California, USA). After blocking with 5% nonfat milk for 2 h, the membranes were incubated with the primary antibody overnight at 4 °C, and followed by subsequently incubation with respective secondary antibodies for 2 h at room temperature. After 3 times washing with TBST, the blots were visualized by electrochemiluminescence plus reagent (Invitrogen, Carlsbad, USA). Finally, the intensity of these blots were quantifed with Image Lab 3.0 software (Bio-Rad, California, USA).

### Immunofluorescence

Chondrocytes were washed in PBS, fixed in 4% paraformaldehyde and permeated in 0.1% Triton X-100 for 15 min. Then the cells were blocked with 5% bovine serum albumin for 1 h at 37 °C, rinsed with PBS and incubated with primary antibodies in a humid chamber overnight at 4 °C. The cells were washed and incubated with Alexa Fluor 488 or Alexa Fluor 594 conjugated second antibodies for 1 h at room temperature and labeled with DAPI for 5 min. Images of each slides were obtained randomly with a fluorescence microscope (Olympus Inc., Tokyo, Japan). Images for co-localization analysis (percentage of co-localization LC3/LAMP1) were assessed using the JaCoP plugin in ImageJ after thresholding of individual frames^52^. All colocalization calculations were performed on five independent experiments with 50 cells per condition in each experiment. Images were prepared for presentation using Adobe Photoshop 6.0 (San Jose, CA, USA).

The immunofluorescence staining was also performed in a vivo study. Following dehydrated and embedded in paraffin, the tissues were cut into 5-µm sagittal sections. For immunofluorescence, sections were deparaffinized in xylene, and rehydrated by ethanol washes. And, sections were incubated with 10% bovine serum albumin for 1 h at room temperature in PBS containing Triton X-100. They were then incubated with primary antibodies overnight at 4 °C in the PBST. After primary antibody incubation, sections were washed for four times for 10 min at room temperature and then incubated with Alexa Fluor 488 Goat anti-rabbit secondary antibody for 1 h at room temperature. Sections were rinsed three times with PBS and incubated with 4,6-diamidino-2-phenylindole (DAPI) for 10 min and finally washed in PBS and sealed with a coverslip. The images were captured with a fluorescence microscope (Olympus Inc., Tokyo, Japan), the rate of TFEB nuclear positive cells each section was quantitated by observers who were blinded to the experimental groups. Five mice of each group were used for quantitative analysis.

### Animal model

Sixty 10-week-old C57BL/6 male wild-type (WT) mice were purchased from Animal Center of Chinese Academy of Sciences Shanghai, China. The mouse osteoarthritis model was induced by surgical destabilization of the medial meniscus (DMM) as previously described^53^. In brief, the mice were anesthetized with intraperitoneally injection of 2% (w/v) pentobarbital (40 mg/kg,) then the joint capsule of right knee was incised just medial to the patellar tendon and the medial meniscotibial ligament was transected with microsurgical scissors. Surgery consisting of an arthrotomy without the transaction of medial meniscotibial ligament, was also performed in the left knee joint of mice and the joint was used as sham group. After surgery, the mice were divided into three groups: Sham + LV-NC, DMM + LV-NC and DMM + LV-TFEB group. Intra-articular injection of 10 μL Lentivirus was performed through a trans-patellar tendon approach at 0, 15, 30 and 45 days post OA surgery. Sham + LV-NC and DMM + LV-NC groups accepted 10 μL LV-NC while DMM + LV-TFEB group was injected with LV-TFEB. Mice were sacrifced at 8 weeks post-OA surgery from each group, the knee joints were dissected and processed for histological evaluation.

### X-ray imaging method

After 8 weeks of surgery within or not treatment, the animals were given the X-ray examination. X-ray imaging was performed on all mice to evaluate the joint space, osteophyte formation and calcification changes of cartilage surface using a digital X-ray machine (Kubtec Model XPERT.8; KUB Technologies Inc.). Proper images were obtained in the following settings: 50 Kv and 160 µA.

### Histopathologic analysis

Slides of each joint were stained with safranin O-fast green (S–O). The cellularity and morphology of cartilage and subchondral bone were examined by another group of experienced histology researchers in a blinded manner using a microscope, and evaluated by using an Osteoarthritis Research Society International (OARSI) scoring system for medial femoral condyle and medial tibial plateau as described previously^54^. The severity of synovitis was graded using a scoring system as previously described^55^. Fifteen mice each group were used for histomorphometric scoring.

### Immunohistochemical analysis

The sections embedded in paraffin were deparaffinized and rehydrated and endogenous peroxidase was blocked by 3% hydrogen peroxide. The sections were incubated with 0.4% pepsin (Sangon Biotech, Shanghai, China) in 5 mM HCl at 37 °C for 20 min for antigen retrieval. The sections were incubated with 5% bovine serum albumin for 30 min at room temperature, then with primary antibody overnight at 4 °C, and finally with HRPconjugated secondary antibody. The rate of positive cells each section was quantitated by observers who were blinded to the experimental groups. Five mice of each group were used for quantitative analysis.

### Statistical analysis

The results were presented as mean ± S.D. Statistical analyses were performed using SPSS statistical software program 20.0 (IBM, Armonk, NY, USA). Data were analyzed by one-way analysis of variance (ANOVA) followed by Tukey’s test for comparison between control and treatment groups. Nonparametric data (OARSI scores and synovitis scores) were analyzed by the Kruskal–Wallis H test. *P* < 0.05 was considered significant.

## Electronic supplementary material

Figure S1

Figure S2

Figure S3

Figure S4

Figure S5

Supplementary figure legends
