# Supplementary figures and images for: TFEB, a potential therapeutic target for osteoarthritis via autophagy regulation
Source: Cell Death Dis. 2018 Aug 28;9(9):858. doi: 10.1038/s41419-018-0909-y (PMC6113230; doi:10.1038/s41419-018-0909-y)

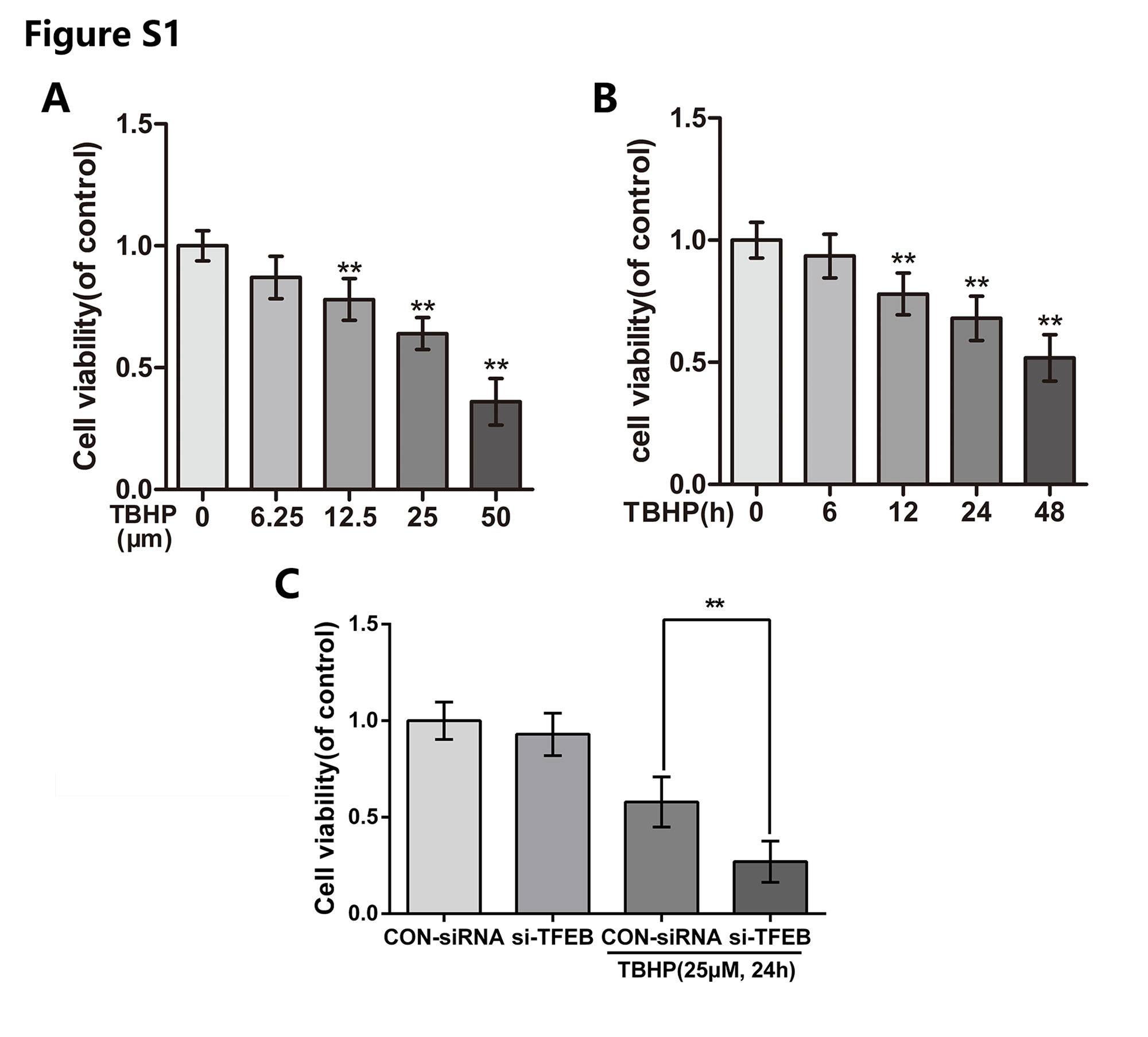

Supplement: Supplementary file 1 — Figure S1 [file 41419_2018_909_MOESM1_ESM.tif]

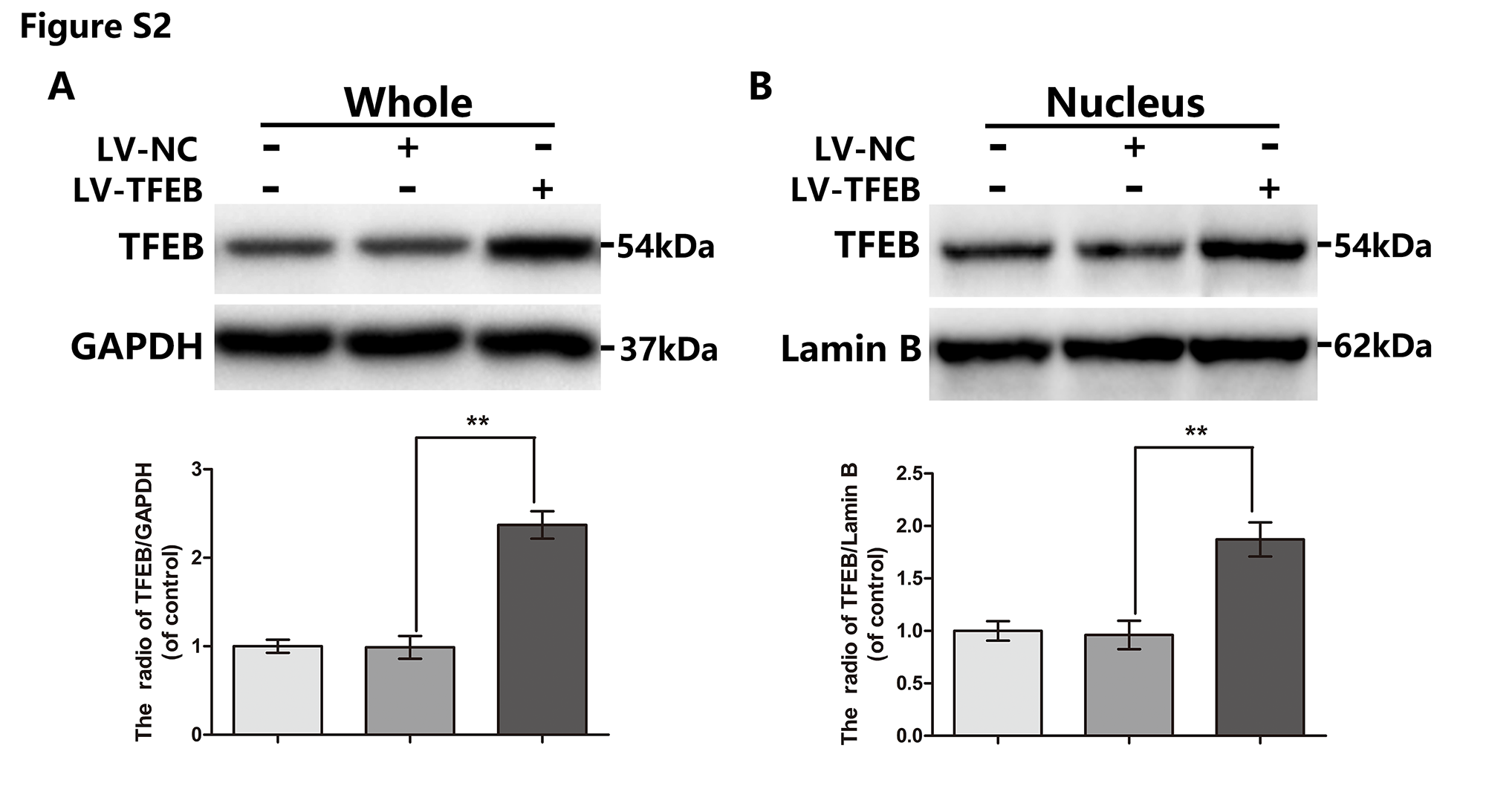

Supplement: Supplementary file 2 — Figure S2 [file 41419_2018_909_MOESM2_ESM.tif]

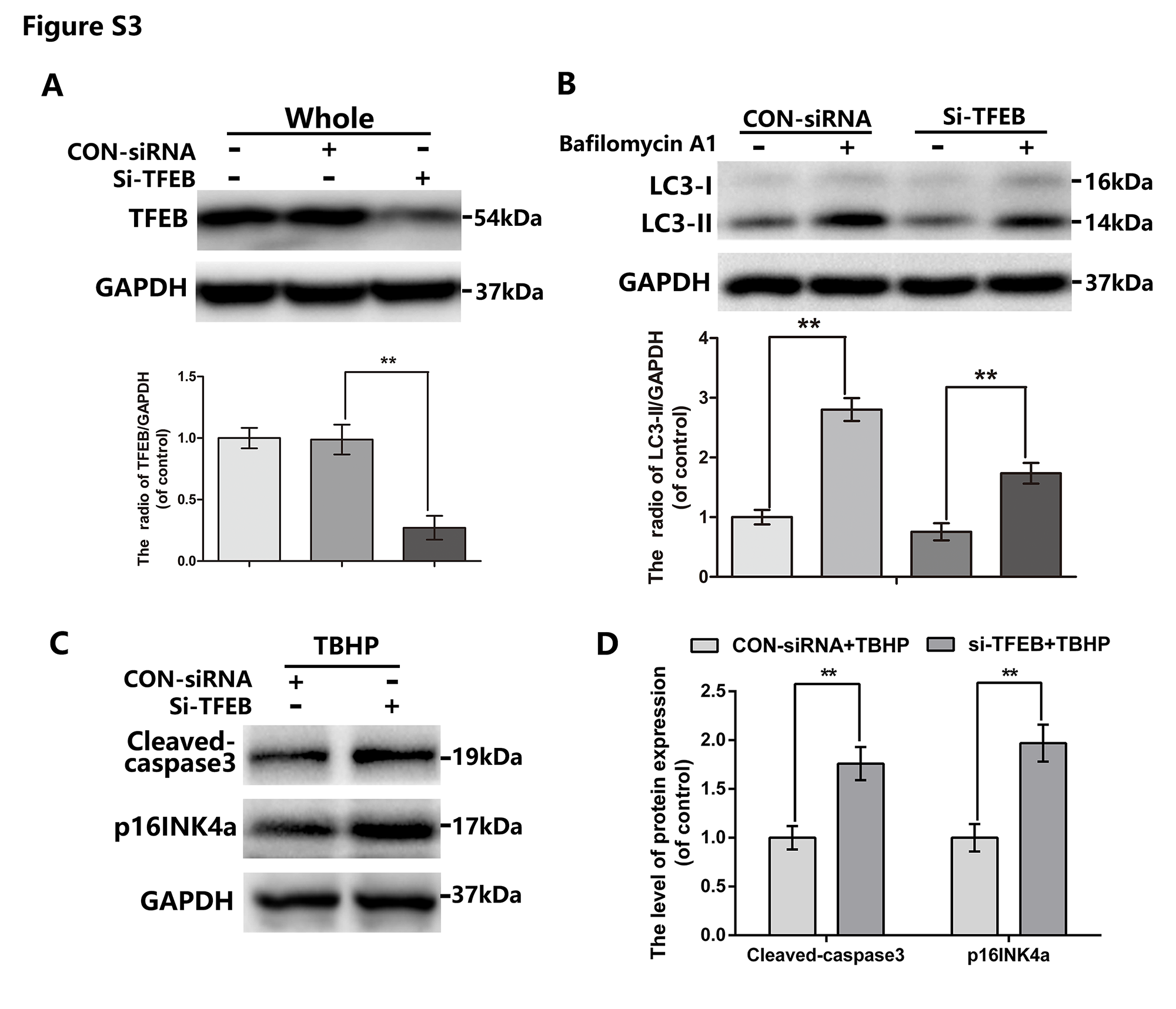

Supplement: Supplementary file 3 — Figure S3 [file 41419_2018_909_MOESM3_ESM.tif]

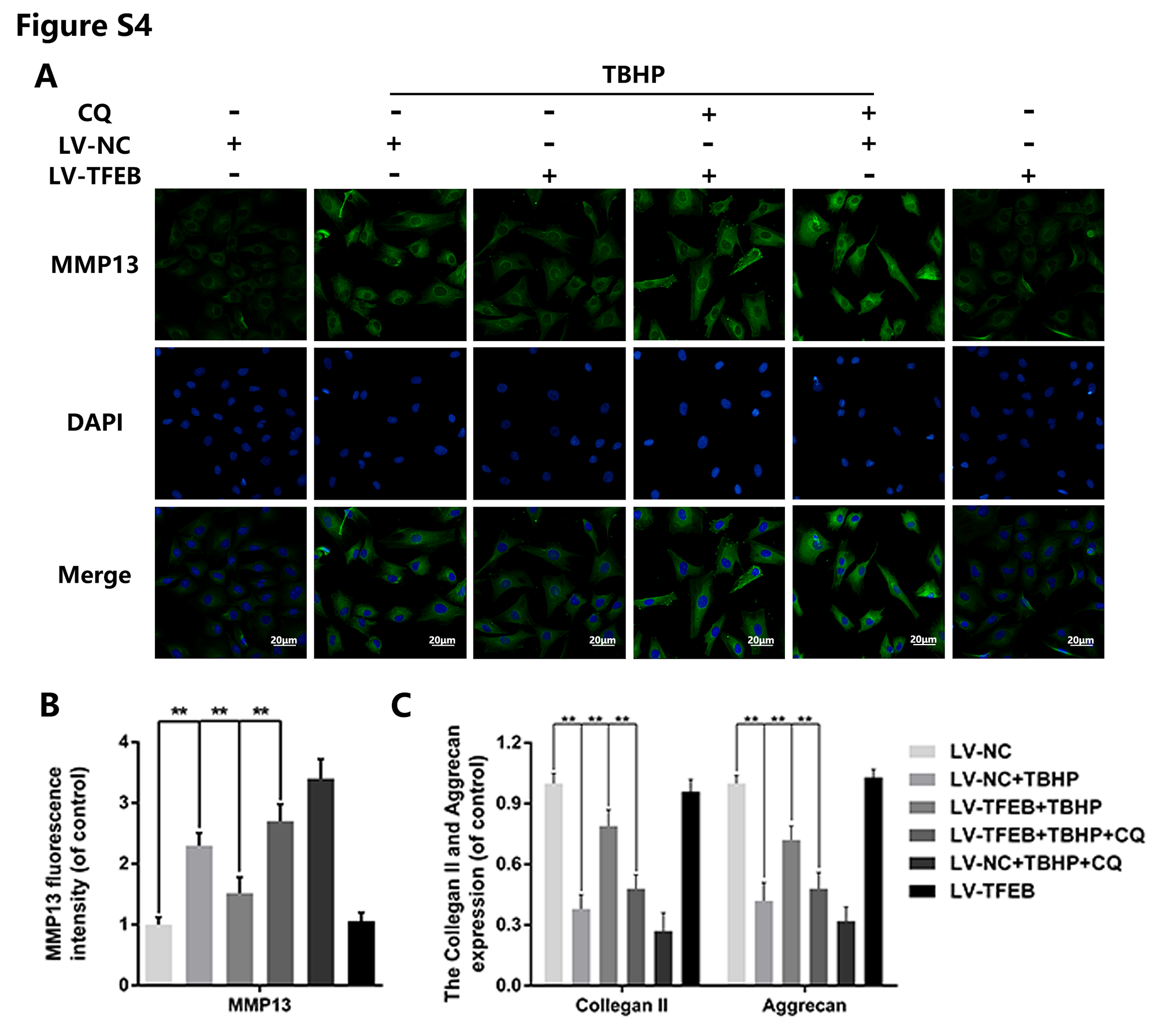

Supplement: Supplementary file 4 — Figure S4 [file 41419_2018_909_MOESM4_ESM.tif]

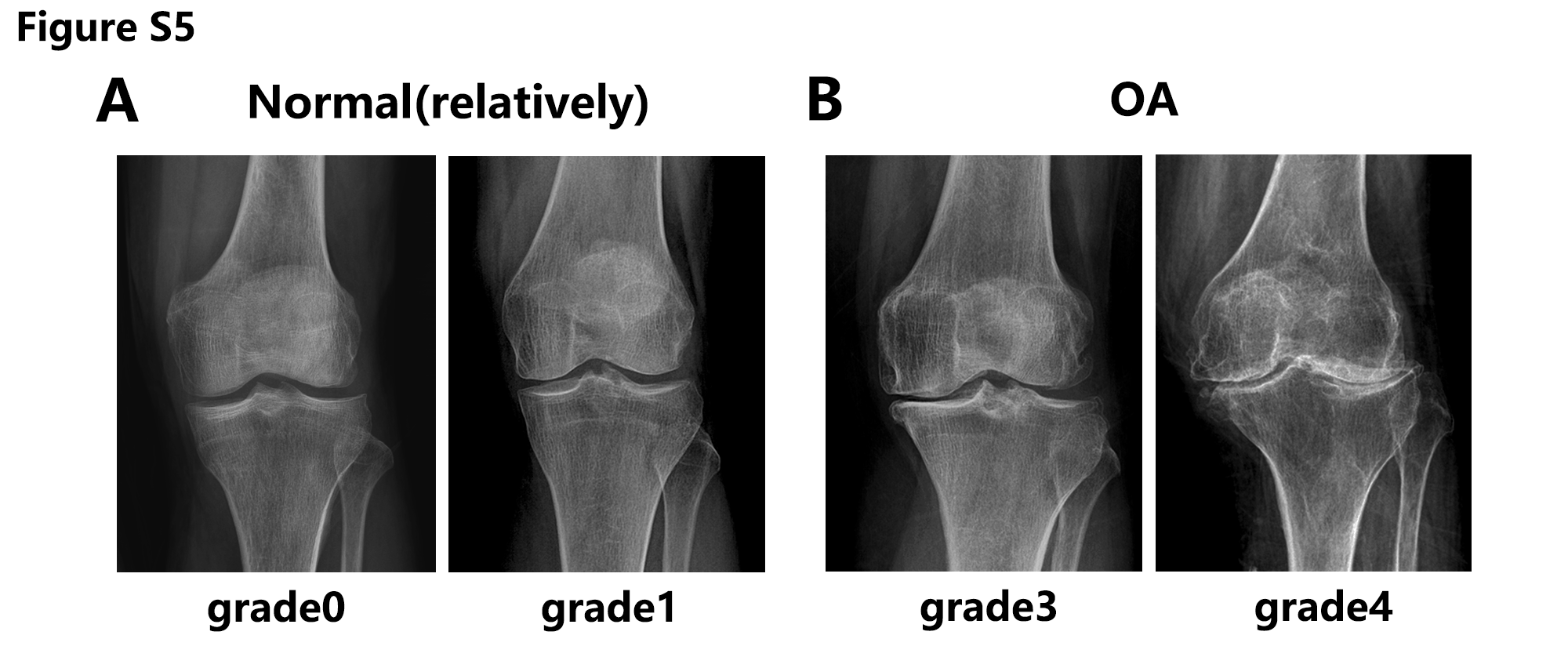

Supplement: Supplementary file 5 — Figure S5 [file 41419_2018_909_MOESM5_ESM.tif]
