# Supplementary material for: TFEB, a potential therapeutic target for osteoarthritis via autophagy regulation
Source: Cell Death Dis. 2018 Aug 28;9(9):858. doi: 10.1038/s41419-018-0909-y (PMC6113230; doi:10.1038/s41419-018-0909-y)
Supplement: Supplementary file 6 — Supplementary figure legends [file 41419_2018_909_MOESM6_ESM.docx]

**Supplementary figure legends**

**Figure S1 The viability of chondrocytes after TBHP treatment with different concentration and time.**

(**A** and **B**) CCK8 was performed to detect cellular viability in mouse chondrocytes treated as above. (**C**) Knockdown of TFEB inhibited the viability of chondrocytes after TBHP treatment. All data represent mean ± S.D. (n = 5). ***P*<0.01.

**Figure S2 The LV-TFEB induced successful overexpression and activation of TFEB in chondrocytes.**

(**A** and **B**) The protein expression of TFEB in mouse chondrocytes treated as above. All data represent mean ± S.D. (n = 5). ***P*<0.01.

**Figure S3 TFEB knockdown decreases the autophagic flux in chondrocytes.**

(**A** and **B**) The protein expression of TFEB and LC3 in mouse chondrocytes treated as above. (**C** and **B**) The protein expression of cleaved-caspase3 and p16INK4a in mouse chondrocytes treated as above. All data represent mean ± S.D. (n = 5). ***P*<0.01.

**Figure S4 TFEB-induced autophagy participates in ECM metabolism in chondrocytes under oxidant stress.**

**(A** and **B)** The MMP13 immunofluorescence staining in mouse chondrocytes treated as above (bar: 20 μm). **(C)** The expression of collagen II and aggrecan were measured by ELISA in mouse chondrocytes treated as above. All data represent mean ± S.D. (n = 5). ***P*<0.01.

**Figure S5 X-ray image of human knee joint with different Kellgren-Lawrence grades.**

(**A**) X-ray image of human knee joint with Kellgren-Lawrence grade 0 or 1 were defined as relative human normal group. (**B**) X-ray image of human knee joint with Kellgren-Lawrence grade 3 or 4 were defined as human OA group.
